# Comparative analyses of downstream signal transduction targets modulated after activation of the AT_1_ receptor by two β-arrestin-biased agonists

**DOI:** 10.3389/fphar.2015.00131

**Published:** 2015-07-01

**Authors:** Geisa A. Santos, Diego A. Duarte, Lucas T. Parreiras-e-Silva, Felipe R. Teixeira, Rafael Silva-Rocha, Eduardo B. Oliveira, Michel Bouvier, Claudio M. Costa-Neto

**Affiliations:** ^1^Department of Biochemistry and Immunology, Ribeirao Preto Medical School, University of São PauloRibeirão Preto, Brazil; ^2^Department of Genetics and Evolution, Federal University of São CarlosSão Carlos, Brazil; ^3^Department of Cellular and Molecular Biology, Ribeirao Preto Medical School, University of São PauloRibeirão Preto, Brazil; ^4^Department of Biochemistry and Molecular Medicine, University of MontrealMontreal, QC, Canada; ^5^Center for Integrative Systems Biology (CISBi), Ribeirao Preto Medical School, University of São PauloRibeirão Preto, Brazil

**Keywords:** GPCR, signal transduction, biased agonism, medicinal chemistry, kinases

## Abstract

G protein-coupled receptors (GPCRs) are involved in essentially all physiological processes in mammals. The classical GPCR signal transduction mechanism occurs by coupling to G protein, but it has recently been demonstrated that interaction with β-arrestins leads to activation of pathways that are independent of the G protein pathway. Also, it has been reported that some ligands can preferentially activate one of these signaling pathways; being therefore called biased agonists for G protein or β-arrestin pathways. The angiotensin II (AngII) AT_1_ receptor is a prototype GPCR in the study of biased agonism due to the existence of well-known β-arrestin-biased agonists, such as [Sar^1^, Ile^4^, Ile^8^]-AngII (SII), and [Sar^1^, D-Ala^8^]-AngII (TRV027). The aim of this study was to comparatively analyze the two above mentioned β-arrestin-biased agonists on downstream phosphorylation events and gene expression profiles. Our data reveal that activation of AT_1_ receptor by each ligand led to a diversity of activation profiles that is far broader than that expected from a simple dichotomy between “G protein-dependent” and “β-arrestin-dependent” signaling. We observed clusters of activation profiles common to AngII, SII, and TRV027, as well as downstream effector activation that are unique to AngII, SII, or TRV027. Analyses of β-arrestin conformational changes after AT_1_ receptor stimulation with SII or TRV027 suggests that the observed differences could account, at least partially, for the diversity of modulated targets observed. Our data reveal that, although the categorization “G protein-dependent” vs. “β-arrestin-dependent” signaling can be of pharmacological relevance, broader analyses of signaling pathways and downstream targets are necessary to generate an accurate activation profile for a given ligand. This may bring relevant information for drug development, as it may allow more refined comparison of drugs with similar mechanism of action and effects, but with distinct side effects.

## Introduction

G protein coupled receptors (GPCRs) represent the largest family of transducers of signals, with more than 800 genes in the human genome. These receptors bear the conserved general structure of seven alpha-helices spanning the cytoplasmic membrane, and for this reason are also referred to as seven transmembrane domain receptors (7TMRs) (Selbie and Hill, 1998; Katritch et al., 2013). GPCRs can be activated by a diversity of ligands, such as small monoamines, peptides, large proteins, and even by light in the case of opsins (Heng et al., 2013). Although the GPCR name was given after their classical mechanism of action that involves interaction with and activation of heterotrimeric G proteins, it is now well accepted that GPCRs can also interact with other effectors which in some cases trigger G protein-independent signaling pathways (Ferrand et al., 2005; Cattaneo et al., 2014). For instance, although β-arrestins were classically considered to only promote GPCR desensitization and internalization, therefore leading to G protein signaling termination (Ferguson et al., 1996), it is now recognized that arrestins also act as scaffolding proteins and organize other effectors including kinases that can trigger G protein-independent signaling pathways (Luttrell et al., 2001; Charest and Bouvier, 2003; Beaulieu et al., 2005; Lefkowitz and Shenoy, 2005). More recently, it has been shown that some ligands bear the property of preferentially triggering one or another pathway, leading to the concepts of “biased agonists” and “functional selectivity” (Azzi et al., 2003; Lefkowitz and Shenoy, 2005; Galandrin et al., 2007; Kenakin, 2007; Drake et al., 2008; Shukla et al., 2008; Kahsai et al., 2011; Audet and Bouvier, 2012). The AT_1_ receptor is a prototype GPCR in the study of biased agonism, and the peptide [Sar^1^, Ile^4^, Ile^8^]-AngII (SII) was one of the first β-arrestin-biased ligands to be described (Holloway et al., 2002). More recently, the company Trevena developed additional β-arrestin-biased ligands for AT_1_ receptor (Violin et al., 2010; Rajagopal et al., 2011; Monasky et al., 2013) including the peptide [Sar^1^, D-Ala^8^]-AngII (TRV027), that is now entering phase 2 clinical trials for the treatment of heart failure (Violin et al., 2014). Both SII and TRV027 are described as preferentially triggering β-arrestin-dependent signaling pathways over the Gq pathways.

**Figure 1:**
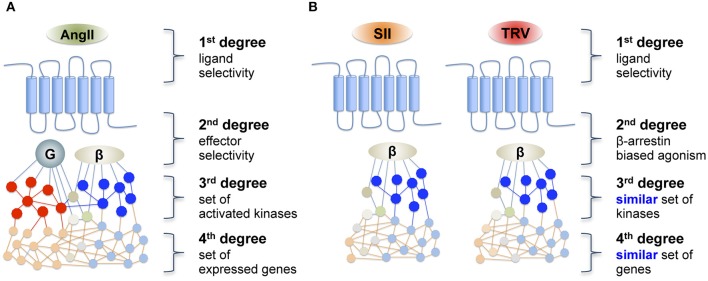
**Schematic representation of (A) a reference agonist and (B) the hypothesis of downstream transduction of a “biased message” after receptor activation by a biased agonist**. AngII, angiotensin II; SII, [Sar^1^, Ile^4^, Ile^8^]-AngII; TRV, TRV027 ([Sar^1^, D-Ala^8^]-AngII).

Based on such different profiles of proximal effector activation when comparing the endogenous ligand, AngII, with β-arrestin-biased ligands such as SII and TRV027, one could hypothesize that downstream signaling cascades, including kinase activation and gene transcription, may be a direct reflection of this dichotomic activation profile. Accordingly, it could be predicted that two β-arrestin-biased ligands would share a similar, but distinct set of downstream signaling events when compared with the endogenous ligands that activate both β-arrestin and Gq as illustrated in Figure 1. To test this hypothesis, we analyzed downstream signals at two distinct levels of the signaling cascade; namely kinase activation and gene transcription. For this purpose, the phosphorylation level of different kinase substrates was assessed using a phosphorylation kinase array profiler, whereas the downstream gene transcription effects were monitored using PCR arrays.

Contrary to the hypothesis that β-arrestin-biased ligands would share downstream signaling signatures that would be distinct from that of AngII, we did not observe more similarities between the two β-arrestin-biased agonists, SII and TRV027, than between each of them and AngII. These results indicate that additional differences in the signaling repertoire of the ligands exist that cannot be captured only by their propensity to differentially activate β-arrestin and Gq-mediated signaling. Such differences may include the differential engagement of other G protein subunits or additional poorly characterized signaling pathways as well as ligand-specific conformational rearrangements of β-arrestin that can result in different downstream responses (Shukla et al., 2008; Zimmerman et al., 2012). Consistent with the latter possibility, we observed distinct conformational rearrangement of β-arrestin promoted by SII and TRV027.

## Materials and methods

### Solid-phase peptide synthesis

SII and TRV027 were synthesized by solid phase synthesis (Merrifield, 1963) using the Fmoc strategy as described before (Fields and Noble, 1990). Briefly, the N-terminal protecting group was removed with 20% piperidine. Amino acids were activated by HOBt/HBTU 0.45 M and couplings were carried-out in the presence of DIPEA 1.2 M and cycles of microwave irradiation. Deprotection and coupling were monitored using the 1% TNBS test. After cleavage from the resin with 90% TFA, peptides were purified by HPLC and the efficiency of synthesis was evaluated by amino acid composition analysis.

### Cell culture and transfection

HEK293T cells were cultured in DMEM supplemented with 10% fetal bovine serum, 100 U/ml penicillin/streptomycin, at 37°C in 5% CO_2_. Cells were seeded in 10-cm dishes, and 48 hours before the experiments were transiently transfected with AT_1_ receptor using the calcium phosphate method (Gether et al., 1993). For BRET experiments, transient transfections were performed using polyethylenimine (PEI; 25 kDa linear; Polysciences, Warrington, PA, USA) at a ratio of 3:1 PEI/DNA, where 5 μg of AT_1_ receptor and 100 ng of RlucII-βarr1-GFP_10_ or RlucII-βarr2-GFP_10_ were cotransfected. The total amount of DNA transfected in each plate was adjusted to 10 μg with salmon sperm DNA (Invitrogen, Carlsbad, CA, USA).

### Kinase phosphorylation array and quantitative analysis

The kinase phosphorylation array (Human Phospho-Kinase Array, Proteome Profiler, R&D Systems, Minneapolis, MN, USA) was used as described before (Souza and Costa-Neto, 2012). Briefly, AT_1_-transfected HEK293T cells were incubated for 16 h in serum-free DMEM prior to stimulation for 10 min with AngII 100 nM, SII 30 μM, TRV027 100 nM, or with vehicle as control. Agonist concentrations were chosen based on their differences in affinities, where SII has lower affinity as compared to AngII and TRV027. Cells were then rinsed, lysed with Lysis Buffer 6 (R&D Systems) and mixed for 30 min at 4°C. The lysates were clarified by centrifugation at 14,000 × g for 5 min at 4°C, and 300 μg of total protein from the lysates was incubated with a pre-blotted membrane array. After lysates were incubated, Chemi Reagent Mix (R&D Systems) was added to the phospho-kinase array membranes and the chemiluminescent signal was captured by ImageQuant 350 (GE Healthcare, Piscataway, NJ, USA). Data were generated from 3 independent experiments. For the analysis of the phosphorylation level, membranes were scanned and processed using ImageJ software (Schneider et al., 2012). The intensities of the spots were quantified and the levels of positive and negative controls were used to set the minimal and maximal intensity levels. For the analysis, only spots with intensity 2-fold higher than background in at least one experiment were considered.

### PCR array and quantitative analysis

Total RNA was extracted from HEK293T cells transiently expressing the AT_1_ receptor and stimulated with AngII 100 nM, SII 30 μM, TRV027 100 nM or vehicle for 6 h. Agonist concentrations were chosen based on their differences in affinities, where SII has lower affinity as compared to AngII and TRV027. After that, cDNAs were synthesized from 1 μg of total RNA using the RT^2^ First Strand Kit (Qiagen, Hilden, Germany). We used the RT^2^ Profiler PCR Array (Human GPCR Signaling PathwayFinder, Qiagen, Hilden, Germany) to simultaneously evaluate the mRNA levels of 84 genes following stimulation with the above-mentioned ligands, following manufacturer's recommended procedures (Table S2).

For the gene expression analysis, fold-change results from 3 independent experiments of Real Time PCR experiments were clustered using Self Organizing Tree Algorithm (Herrero et al., 2001) implemented in MeV software (Saeed et al., 2003). For this analysis, we considered only genes with a fold-change of ±2.0 in at least one of the experimental conditions assayed.

### BRET assay

HEK293T cells transiently expressing the AT_1_ receptor and RlucII-βarr1-GFP_10_ or RlucII-βarr2-GFP_10_ were washed once with PBS, detached and seeded in 96-well white plates (OptiPlate; PerkinElmer, Waltham, MA, USA). BRET was monitored in a Victor™ X Light Luminescence microplate reader (PerkinElmer) equipped with different donor/acceptor emission filter sets, after addition of 5 μM coelenterazine-400A (Biotium, Hayward, CA, USA) to the cells. BRET signals were derived from the emission detected with the energy acceptor filter (515 ± 15 nm) divided by the emission detected using the energy donor filter (410 ± 40 nm). Five independent experiments with full concentration-response curves and time-course assays were performed in cells stimulated with AngII, SII or TRV027. Concentrations used for time-course assays were AngII 100 nM, SII 30 μM, and TRV027 100 nM. Agonist concentrations were chosen based on their differences in affinities, where SII has lower affinity as compared to AngII and TRV027.

### Statistical analyses

For phosphorylation level assays, only kinase substrates with change in intensities of ±30% that were statistically significant when analyzed by One-Way ANOVA test were considered to be affected by the condition (Table S1), as described previously (Xiao et al., 2010). Conformational change of β-arrestin in BRET assays was analyzed by One-Way ANOVA test and statistical significance was accepted when *P* < 0.05. Data graphs were generated and statistical analyses were performed using GraphPad software (GraphPad, San Diego, CA).

## Results

### Activation of the AT_1_ receptor by AngII, SII, or TRV027 reveals unique patterns of kinase phosphorylation for the different ligands

**Figure 2:**
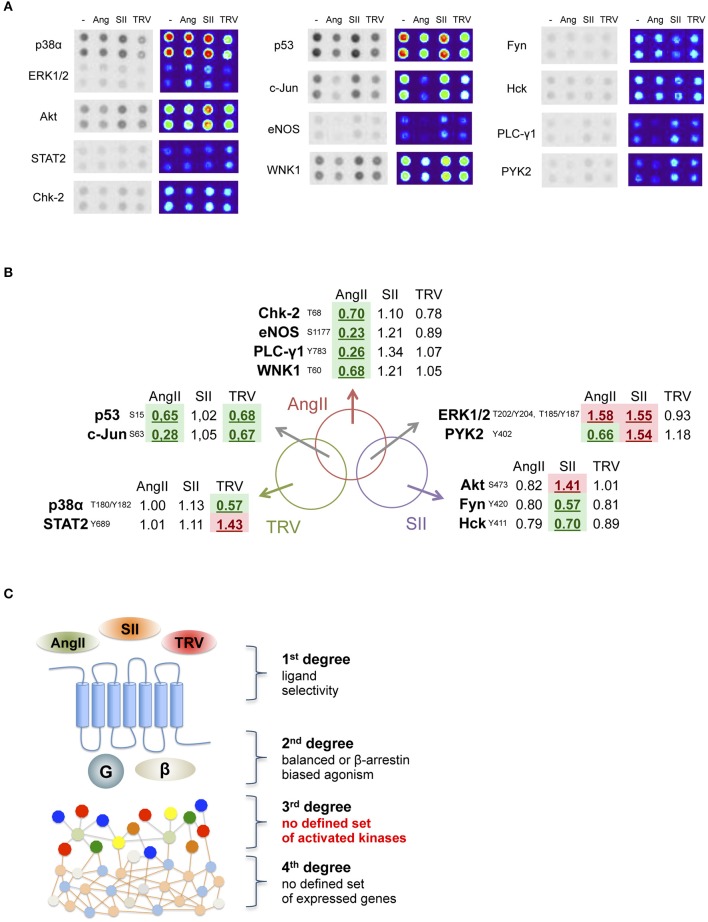
**Analysis of 13 human kinase substrate phosphorylation levels whose phosphorylation status changed after stimulation with AngII, SII or TRV027. (A)** Quantification of kinase substrate phosphorylation levels in response to AngII, and two biased agonists for 10 min. For clarity, spot intensities were converted to a false color scale to facilitate the visualization of the differences. **(B)** Venn diagram representing the set of kinase substrates differentially modulated in the three experimental conditions used. **(C)** Schematic representation of the obtained data, showing that no clear biased message was observed at the level of kinase substrate phosphorylation after stimulation with biased agonists.

We analyzed the phosphorylation pattern of 43 different kinase substrates exposed to lysates from cells transfected with AT_1_ receptor and stimulated with AngII, SII, or TRV027 for 10 min. Figure 2A shows that among 43 kinase substrates analyzed, 13 were phosphorylated or dephosphorylated upon stimulation with the above-listed ligands as compared to vehicle-treated cells (see Table S1 for a list of all kinase substrates included in the array as well as their phosphorylation status after stimulation with AngII, SII, or TRV027). In some cases, we observed that the phosphorylation pattern for some kinase substrates was significantly modulated upon treatment with only one of the tested ligands, AngII (e.g., Chk-2 and eNOS), or with SII (e.g., Akt and Fyn), or with TRV027 (e.g., p38α and STAT2). We also found that the phosphorylation pattern of some kinase substrates was similarly modulated by AngII and either TRV027 (e.g., p53 and c-JUN), or SII (e.g., ERK1/2); but no substrate phosphorylation was similarly modulated by the two β-arrestin-biased agonists TRV027 and SII, as can be seen in the Venn diagram in Figure 2B. These results clearly indicate that the simple β-arrestin bias of the ligand is not sufficient to explain the different kinase substrate phosphorylation patterns between TRV027 and SII (Figure 2C).

### Activation of the AT_1_ receptor by AngII, SII, or TRV027 reveals unique patterns of gene expression

**Figure 3:**
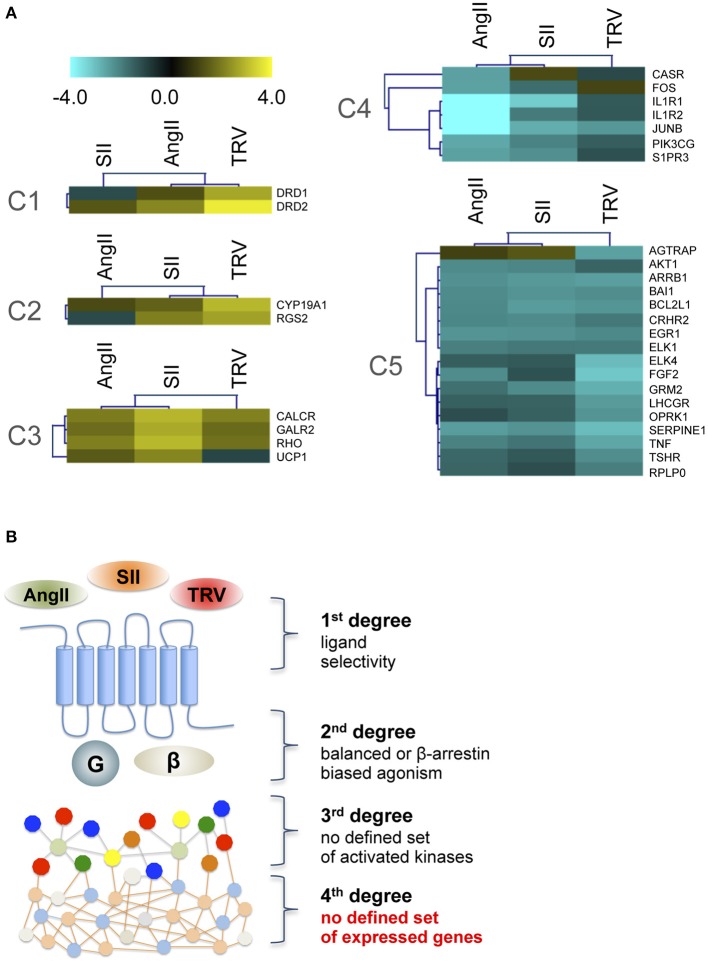
**Cluster analysis of target genes differentially modulated by the three ligands assayed. (A)** The five gene clusters identified using the Self Organizing Tree Algorithm (Herrero et al., 2001). Fold-changes were calculated comparing to untreated samples and are represented on a log scale. **(B)** Schematic representation of the obtained data, showing that no clear biased message was observed at the level of gene expression modulation after stimulation with biased agonists.

To analyze further downstream targets after receptor activation by distinct ligands, we used a PCR array to analyze the expression levels of 84 genes after stimulation with AngII, SII, or TRV027 (see Table S2 for a list of all genes and their mRNA levels after stimulation with the different ligands). Similarly to what we observed with the phosphorylation kinase array, no common patterns of expression modulation could be correlated to the β-arrestin engagement bias (Figure 3). Indeed, the set of modulated genes after stimulation with the reference agonist, AngII, were not clearly distinct from the set of modulated genes after stimulation with the β-arrestin-biased agonists. Remarkably, we also found no strong overlap between the sets of genes modulated by the two β-arrestin-biased agonists. Based on the PCR array data, we generated clusters of patterns for modulated genes. For this, we used Self-Organizing Tree Algorithm (SOTA), an unsupervised neural network widely used to perform hierarchical clustering of gene expression patterns (Herrero et al., 2001). In this sense, SOTA defines the hierarchical relationship between sets of expression profiles based on appropriate distance functions, thus identifying groups of genes with similar expression profiles (Herrero et al., 2001). Finally, cluster boundaries (i.e., the total number of clusters generated) are self-defined depending on the level of heterogeneity of the expression data. Applying SOTA to the data obtained here (Table S2) yielded clearly distinct patterns for each ligand with greater similarities being observed between AngII and either SII or TRV027 than between SII and TRV027, with a total of five clusters being identified (Figure 3). Among the differences between SII and TRV027, UCP1 was found to be negatively modulated by TRV027 while being positively regulated by SII (Figure 3A, cluster C3), and AGTRAP, an AT_1_ receptor associated protein, was negatively modulated by TRV027 but not affected by SII (Figure 3A, cluster C5). Only two genes were similarly modulated by SII and TRV027 (Figure 3A, cluster C2), one of them being the negative regulator of G protein signaling, RGS2.

As observed for the kinase phosphorylation array data, the gene expression data indicate that the activation of the receptor by β-arrestin-biased agonists does not lead to a similar set of modulated genes suggesting other sources of signaling bias.

### AngII, SII, and TRV027 promote distinct conformational changes of β-arrestins

To assess whether the differences in downstream signaling of TRV027 and SII could result from differential conformational rearrangements of β-arrestin as previously shown for other AT_1_ receptor ligands (Shukla et al., 2008; Zimmerman et al., 2012; Gurevich and Gurevich, 2014), we used a BRET-based biosensor that monitors the conformational rearrangement of β-arrestins by measuring intra-molecular BRET between RLucII and GFP_10_ fused to the N- and C-termini of β-arrestins respectively (RlucII-βarrestin-GFP_10_) (Charest et al., 2005). Using this approach, the amplitude of BRET changes provides an indirect reflection of the conformational rearrangement of β-arrestin upon its recruitment to the receptor promoted by a given ligand.

**Figure 4:**
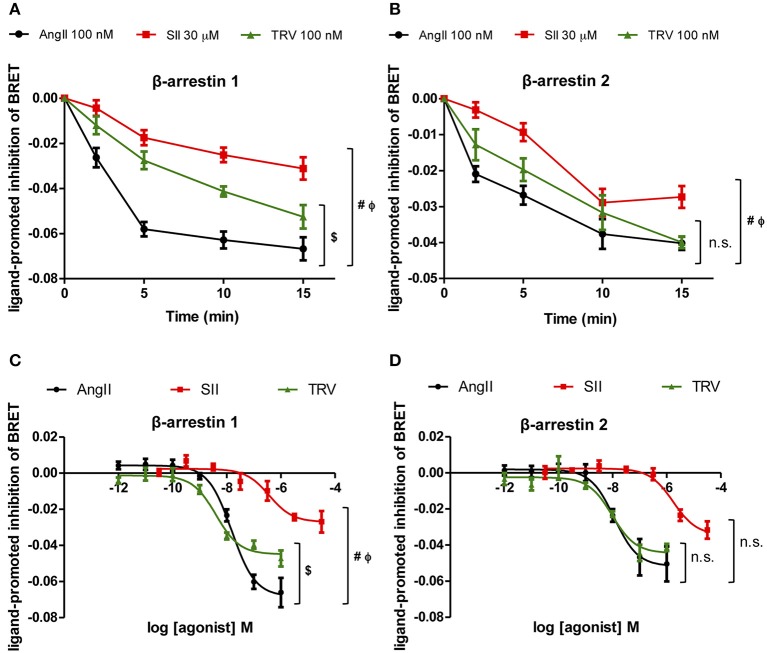
**Analyses of β-arrestin-1 and -2 conformational changes after stimulation with the three ligands assayed**. Kinetics of conformational changes for β-arrestin-1 **(A)** and β-arrestin-2 **(B)** were evaluated using a fixed concentration of each ligand, the same concentrations used in the kinase phosphorylation array and the PCR array. Concentration-response curves were performed after 10 min stimulation and conformational changes were analyzed for β-arrestin-1 **(C)** and 2 **(D)**. One-Way ANOVA, *p* = 0.05: (**#**) AngII vs. SII; ($) AngII vs. TRV; (Φ) SII vs. TRV; (n.s.) non-significant. One-Way ANOVA was performed using the area under the curve **(A,B)** or the curve bottom plateaus **(C,D)**.

To test the effect of the different AT_1_ receptor ligands on the conformational changes of βarrestin-1 and -2, time-courses of the ligand-promoted changes in BRET were monitored in cells co-expressing the AT_1_ receptor and either RlucII-βarrestin1-GFP_10_ or RlucII-βarrestin2-GFP_10_ and stimulated with AngII, SII, or TRV027 at the same concentrations as used in the kinase substrate phosphorylation and qPCR arrays. As shown in Figures 4A,B, the 3 ligands induced a time-dependent decrease in BRET for both β-arrestin-1 and -2, consistent with their ability to promote the recruitment of the two β-arrestins to AT_1_ receptor and the ensuing conformational change. However, the kinetics of the changes was shown to be distinct for the 3 ligands, mainly for βarrestin-1, with AngII yielding the fastest decrease followed by TRV027 and then SII. Even more striking is the fact that although the decreases reached the same level for AngII and TRV027 at 15 min, it was significantly higher for SII both for β-arrestin-1 and β-arrestin-2. These data clearly point to distinct β-arrestin conformational rearrangements upon stimulation with the 3 ligands within the experimental time of the assay. To reinforce these findings, we have performed concentration-response curves (Figures 4C,D) that fully corroborate the kinetics data. As seen in the time-course assays, differences for β-arrestin-1 conformational changes were more pronounced then those observed for β-arrestin-2 (Figures 4C,D).

## Discussion

The results reported here show that the complexity of signaling pathways activated by AT_1_ receptor, and possibly all GPCRs, is greater than can be explained only by the bias of ligands toward β-arrestin. Based on our findings from kinase substrate phosphorylation and gene expression arrays (respectively, Figures 2, 3), we hypothesize that the diversity of activated targets in the two levels studied could be, at least partially, due to the diversity of β-arrestin conformations that can be stabilized after receptor stimulation by the different ligands. Such distinct conformations would be responsible for stabilizing different complexes that have arrestins as scaffold proteins, and therefore would lead to distinct signaling cascades. It has been reported that β-arrestins adopt different conformations after AT_1_ receptor activation depending on the stimulus and the phosphorylation state of the receptor (Shukla et al., 2008). Another study showed that the distinct conformations adopted by β-arrestin, depending on the ligand used to activate AT_1_ receptor, would be responsible for stabilizing different complexes having arrestins as scaffold proteins, and therefore would lead to distinct signaling cascades with profound impact on physiological and cellular processes (Zimmerman et al., 2012). Here, kinetic conformational assays suggest that SII and TRV027 might induce different conformations of both arrestins depending on the time of stimulation, where a clearer difference was observed for β-arrestin-1 (see Figures 4A,B). It is interesting to note that β-arrestin-2 is commonly reported as the main arrestin involved in AT_1_ receptor signaling (Hunton et al., 2005; Rajagopal et al., 2006; Ahn et al., 2009), while here we have observed a more prominent conformational change for β-arrestin-1. We cannot exclude that the observed effects could result in part from different on-rates or off-rates of the ligands. For instance, despite AngII and TRV027 having similar affinities, the observed effects may result from a combination of distinct on- and off-rates, which in turn could lead to different kinetics of β-arrestin conformational changes. Considering the complexity of the cellular environment and longer exposition periods in some situations, we believe that such different sets of conformations may account, at least partially, for the diversity of responses we found in this study as shown in Figures 2, 3.

Another possible explanation for our results that cannot be ruled out is that activation of the AT_1_ receptor by the studied ligands may comprise other unknown effectors. If this is the case, one could speculate that two β-arrestin-biased agonists such as SII and TRV027, despite leading to a preferential coupling to arrestins, might induce coupling to distinct “third” and “fourth” effectors, therefore introducing a diversification in the signaling pathways.

It has been shown that AngII and SII are able to activate many subtypes of G proteins, namely G_i1_, G_i2_, G_i3_, G_oA_, G_oB_, G_q_, G_11_, and G_13_, to different extents after activation of AT_1_ receptor, which account for different signaling outputs of the ERK1/2 pathway (Sauliere et al., 2012). Also, AT_1_ receptor is described to trigger several signaling pathways such as PI3K/Akt, JAK/STAT, transactivation of receptor tyrosine kinases such as EGFR and activation of non-receptor tyrosine kinases such as Src, Pyk2, and FAKs (Marrero et al., 1997; Tang et al., 2000; Jiang et al., 2007; Costa-Neto et al., 2014). As it is still necessary to evaluate how and to what extent biased ligands may differentially activate the diversity of G protein subunits and regulate the aforementioned pathways, we speculate that it could account, at least partially, for the different profiles of kinase substrate phosphorylation and gene expression levels reported in this study.

While such remarks surely deserve further analyses, we believe that our data unveil the extreme complexity of signaling cascades that can be modulated, even after activation by supposedly “more selective” agonists. This observation denotes no demerit to the concept of biased agonism, which we believe is of extreme importance to development of future drugs, but rather highlights the need for thorough and comprehensive analyses when characterizing novel ligands and signaling pathways.

### Conflict of interest statement

The authors declare that the research was conducted in the absence of any commercial or financial relationships that could be construed as a potential conflict of interest.
