# Supplementary material for: Comparative analyses of downstream signal transduction targets modulated after activation of the AT1 receptor by two β-arrestin-biased agonists
Source: Front Pharmacol. 2015 Jul 1;6:131. doi: 10.3389/fphar.2015.00131 (PMC4486767; doi:10.3389/fphar.2015.00131)
Supplement: Supplementary file 1 [file Table1.DOCX]

**Table S1.** Phosphorylation profiles of the analyzed kinase substrates.

| Protein | Site (~P) | Spot | Control | AngII | SII | TRV |
| --- | --- | --- | --- | --- | --- | --- |
| Reference | - | A1.2 | 1.00 | 1.00 | 0.88 | 0.98 |
| p38α | T180/Y182 | A3.4 | 1.00 | 1.00 | 1.13 | 0.57 |
| ERK1/2 | T202/Y204.T185/Y187 | A5.6 | 1.00 | 1.58 | 1.55 | 0.93 |
| JNK pan | T183/Y185. T221/Y223 | A7.8 | 1.00 | 1.01 | 1.26 | 0.97 |
| ^#^GSK-3α/β | S21/S9 | A9.10 | 1.00 | 1.11 | 1.53 | 1.35 |
| EGF R | Y1086 | B3.4 | 1.00 | 1.02 | 1.14 | 0.91 |
| MSK1/2 | S376/S360 | B5.6 | 1.00 | 1.01 | 1.14 | 0.86 |
| AMPKα1 | T174 | B7.8 | 1.00 | 1.03 | 1.34 | 1.10 |
| Akt | S473 | B9.10 | 1.00 | 0.82 | 1.41 | 1.01 |
| TOR | S2448 | C1.2 | 1.00 | 0.96 | 0.97 | 1.01 |
| CREB | S133 | C3.4 | 1.00 | 1.17 | 1.19 | 0.87 |
| HSP27 | S78/S82 | C5.6 | 1.00 | 0.79 | 1.03 | 1.10 |
| AMPKα2 | T172 | C7.8 | 1.00 | 1.07 | 1.15 | 1.16 |
| β-Catenin | - | C9.10 | 1.00 | 1.06 | 1.57 | 1.13 |
| Src | Y419 | D1.2 | 1.00 | 0.95 | 0.89 | 1.09 |
| Lyn | Y397 | D3.4 | 1.00 | 0.89 | 0.85 | 0.92 |
| Lck | Y394 | D5.6 | 1.00 | 1.09 | 1.19 | 1.30 |
| STAT2 | Y689 | D7.8 | 1.00 | 1.01 | 1.11 | 1.43 |
| STAT5a | Y694 | D9.10 | 1.00 | 0.84 | 1.24 | 0.85 |
| Fyn | Y420 | E1.2 | 1.00 | 0.80 | 0.57 | 0.81 |
| Yes | Y426 | E3.4 | 1.00 | 0.85 | 1.04 | 1.04 |
| Fgr | Y412 | E5.6 | 1.00 | 0.71 | 0.88 | 0.78 |
| STAT6 | Y641 | E7.8 | 1.00 | 0.97 | 1.28 | 1.22 |
| STAT5b | Y699 | E9.10 | 1.00 | 0.98 | 1.03 | 0.83 |
| Hck | Y411 | F1.2 | 1.00 | 0.79 | 0.70 | 0.89 |
| Chk-2 | T68 | F3.4 | 1.00 | 0.70 | 1.10 | 0.78 |
| FAK | Y397 | F5.6 | 1.00 | 0.88 | 1.21 | 1.03 |
| ^#^PDGF Rβ | Y751 | F7.8 | 1.00 | 1.09 | 1.58 | 1.41 |
| STAT5a/b | Y694/Y699 | F9.10 | 1.00 | 0.86 | 1.03 | 0.79 |
| Reference | - | G1.2 | 1.00 | 0.96 | 1.06 | 1.05 |
| PRAS40 | T246 | G3.4 | 1.00 | 0.68 | 0.96 | 0.76 |
| p53 | S392 | A13.14 | 1.00 | 0.92 | 1.01 | 0.76 |
| Reference | - | A17.18 | 1.00 | 0.91 | 0.97 | 0.99 |
| Akt | T308 | B11.12 | 1.00 | 0.84 | 1.13 | 0.96 |
| p53 | S46 | B13.14 | 1.00 | 0.78 | 0.97 | 0.72 |
| p70 S6 Kinase | T389 | C11.12 | 1.00 | 0.84 | 1.12 | 0.88 |
| p53 | S15 | C13.14 | 1.00 | 0.65 | 1.02 | 0.68 |
| c-Jun | S63 | C15.16 | 1.00 | 0.28 | 1.05 | 0.67 |
| p70 S6 Kinase | S6 | D11.12 | 1.00 | 0.83 | 1.02 | 0.97 |
| RSK1/2/3 | S380/S386/S377 | D13.14 | 1.00 | 0.78 | 0.98 | 0.98 |
| eNOS | S1177 | D15.16 | 1.00 | 0.23 | 1.21 | 0.89 |
| STAT3 | Y705 | E11.12 | 1.00 | 0.93 | 1.20 | 1.02 |
| p27 | T198 | E13.14 | 1.00 | 1.02 | 1.75 | 1.71 |
| PLC-γ1 | Y783 | E15.16 | 1.00 | 0.26 | 1.34 | 1.07 |
| STAT3 | S727 | F11.12 | 1.00 | 0.89 | 1.13 | 1.18 |
| WNK1 | T60 | F13.14 | 1.00 | 0.68 | 1.21 | 1.05 |
| PYK2 | Y402 | F15.16 | 1.00 | 0.66 | 1.54 | 1.18 |
| HSP60 | - | G11.12 | 1.00 | 0.82 | 1.04 | 0.93 |

Values appear underlined only when below 0.70 or above 1.30 and statistically significant when compared to the control condition by one-way ANOVA test.

**^#^** In these two cases, values passed the threshold criteria and were statistically significant, but absolute quantification of spot intensity was too low (i.e., less than twice above background values) and did not display a clear profile when analyzed using ImageJ.
