# Supplementary material for: Comparative analyses of downstream signal transduction targets modulated after activation of the AT1 receptor by two β-arrestin-biased agonists
Source: Front Pharmacol. 2015 Jul 1;6:131. doi: 10.3389/fphar.2015.00131 (PMC4486767; doi:10.3389/fphar.2015.00131)
Supplement: Supplementary file 2 [file Table2.DOCX]

**Table S2.** Expression modulation of genes analyzed by PCR array.

| Gene Description | AngII | SII | TRV120027 |
| --- | --- | --- | --- |
|  | Fold change | Fold change | Fold change |
| [Adenylate cyclase 5](http://www.ncbi.nlm.nih.gov/sites/entrez?db=gene&cmd=Retrieve&dopt=Graphics&list_uids=111) | -1.34 | -1.18 | -1.24 |
| [Adenosine A2a receptor](http://www.ncbi.nlm.nih.gov/sites/entrez?db=gene&cmd=Retrieve&dopt=Graphics&list_uids=135) | 1.01 | 1.06 | 1.00 |
| [Adrenergic. beta-1-. receptor](http://www.ncbi.nlm.nih.gov/sites/entrez?db=gene&cmd=Retrieve&dopt=Graphics&list_uids=153) | -1.43 | -1.40 | -1.91 |
| [Adrenergic. beta-2-. receptor. surface](http://www.ncbi.nlm.nih.gov/sites/entrez?db=gene&cmd=Retrieve&dopt=Graphics&list_uids=154) | -1.49 | -1.20 | -1.92 |
| [Angiotensinogen (serpin peptidase inhibitor. clade A. member 8)](http://www.ncbi.nlm.nih.gov/sites/entrez?db=gene&cmd=Retrieve&dopt=Graphics&list_uids=183) | 1.06 | -1.03 | 1.68 |
| [Angiotensin II receptor. type 1](http://www.ncbi.nlm.nih.gov/sites/entrez?db=gene&cmd=Retrieve&dopt=Graphics&list_uids=185) | -1.29 | -1.08 | -1.40 |
| [Angiotensin II receptor. type 2](http://www.ncbi.nlm.nih.gov/sites/entrez?db=gene&cmd=Retrieve&dopt=Graphics&list_uids=186) | -1.07 | -1.12 | 1.25 |
| [Angiotensin II receptor-associated protein](http://www.ncbi.nlm.nih.gov/sites/entrez?db=gene&cmd=Retrieve&dopt=Graphics&list_uids=57085) | -2.48 | -2.33 | -2.97 |
| [V-akt murine thymoma viral oncogene homolog 1](http://www.ncbi.nlm.nih.gov/sites/entrez?db=gene&cmd=Retrieve&dopt=Graphics&list_uids=207) | -2.30 | -2.42 | -2.51 |
| [Arrestin. beta 1](http://www.ncbi.nlm.nih.gov/sites/entrez?db=gene&cmd=Retrieve&dopt=Graphics&list_uids=408) | -2.20 | -2.34 | -2.23 |
| [Arrestin. beta 2](http://www.ncbi.nlm.nih.gov/sites/entrez?db=gene&cmd=Retrieve&dopt=Graphics&list_uids=409) | -1.22 | -1.40 | -1.95 |
| [Brain-specific angiogenesis inhibitor 1](http://www.ncbi.nlm.nih.gov/sites/entrez?db=gene&cmd=Retrieve&dopt=Graphics&list_uids=575) | -2.20 | -2.12 | -1.57 |
| [B-cell CLL/lymphoma 2](http://www.ncbi.nlm.nih.gov/sites/entrez?db=gene&cmd=Retrieve&dopt=Graphics&list_uids=596) | -1.41 | -1.19 | -1.20 |
| [BCL2-like 1](http://www.ncbi.nlm.nih.gov/sites/entrez?db=gene&cmd=Retrieve&dopt=Graphics&list_uids=598) | -2.18 | -2.47 | -2.36 |
| [CALCITONIN RECEPTOR](http://www.ncbi.nlm.nih.gov/sites/entrez?db=gene&cmd=Retrieve&dopt=Graphics&list_uids=799) | 1.99 | 2.89 | 2.07 |
| [Calcitonin receptor-like](http://www.ncbi.nlm.nih.gov/sites/entrez?db=gene&cmd=Retrieve&dopt=Graphics&list_uids=10203) | -1.74 | -1.31 | -1.69 |
| [Calcium-sensing receptor](http://www.ncbi.nlm.nih.gov/sites/entrez?db=gene&cmd=Retrieve&dopt=Graphics&list_uids=846) | -6.84 | -3.23 | -1.39 |
| [Chemokine (C-C motif) ligand 2](http://www.ncbi.nlm.nih.gov/sites/entrez?db=gene&cmd=Retrieve&dopt=Graphics&list_uids=6347) | 1.34 | 1.68 | 1.75 |
| [Chemokine (C-C motif) ligand 4](http://www.ncbi.nlm.nih.gov/sites/entrez?db=gene&cmd=Retrieve&dopt=Graphics&list_uids=6351) | -1.97 | -1.31 | -1.96 |
| [Cyclin D1](http://www.ncbi.nlm.nih.gov/sites/entrez?db=gene&cmd=Retrieve&dopt=Graphics&list_uids=595) | -1.07 | 1.02 | -1.30 |
| [Cyclin E1](http://www.ncbi.nlm.nih.gov/sites/entrez?db=gene&cmd=Retrieve&dopt=Graphics&list_uids=898) | -1.70 | -1.56 | -1.99 |
| [Cyclin E2](http://www.ncbi.nlm.nih.gov/sites/entrez?db=gene&cmd=Retrieve&dopt=Graphics&list_uids=9134) | 1.04 | 1.22 | -1.02 |
| [Cyclin-dependent kinase inhibitor 1A (p21. Cip1)](http://www.ncbi.nlm.nih.gov/sites/entrez?db=gene&cmd=Retrieve&dopt=Graphics&list_uids=1026) | 1.21 | 1.36 | 1.33 |
| [Cyclin-dependent kinase inhibitor 1B (p27. Kip1)](http://www.ncbi.nlm.nih.gov/sites/entrez?db=gene&cmd=Retrieve&dopt=Graphics&list_uids=1027) | -1.65 | -1.38 | -1.57 |
| [CASP8 and FADD-like apoptosis regulator](http://www.ncbi.nlm.nih.gov/sites/entrez?db=gene&cmd=Retrieve&dopt=Graphics&list_uids=8837) | -1.86 | -1.58 | -1.67 |
| [Collagen. type I. alpha 1](http://www.ncbi.nlm.nih.gov/sites/entrez?db=gene&cmd=Retrieve&dopt=Graphics&list_uids=1277) | -1.45 | -1.34 | -1.34 |
| [Corticotropin releasing hormone receptor 1](http://www.ncbi.nlm.nih.gov/sites/entrez?db=gene&cmd=Retrieve&dopt=Graphics&list_uids=1394) | -1.13 | -1.02 | 1.80 |
| [Corticotropin releasing hormone receptor 2](http://www.ncbi.nlm.nih.gov/sites/entrez?db=gene&cmd=Retrieve&dopt=Graphics&list_uids=1395) | -1.63 | -1.38 | -2.23 |
| [Connective tissue growth factor](http://www.ncbi.nlm.nih.gov/sites/entrez?db=gene&cmd=Retrieve&dopt=Graphics&list_uids=1490) | -1.08 | 1.36 | -1.16 |
| [Cytochrome P450. family 19. subfamily A. polypeptide 1](http://www.ncbi.nlm.nih.gov/sites/entrez?db=gene&cmd=Retrieve&dopt=Graphics&list_uids=1588) | 1.17 | 1.54 | 2.91 |
| [Dopamine receptor D1](http://www.ncbi.nlm.nih.gov/sites/entrez?db=gene&cmd=Retrieve&dopt=Graphics&list_uids=1812) | 1.24 | -1.17 | 2.66 |
| [Dopamine receptor D2](http://www.ncbi.nlm.nih.gov/sites/entrez?db=gene&cmd=Retrieve&dopt=Graphics&list_uids=1813) | 2.16 | 1.35 | 3.73 |
| [Dual specificity phosphatase 14](http://www.ncbi.nlm.nih.gov/sites/entrez?db=gene&cmd=Retrieve&dopt=Graphics&list_uids=11072) | -1.76 | -1.62 | -1.93 |
| [Endothelin 1](http://www.ncbi.nlm.nih.gov/sites/entrez?db=gene&cmd=Retrieve&dopt=Graphics&list_uids=1901) | -1.14 | -1.21 | -1.44 |
| [Early growth response 1](http://www.ncbi.nlm.nih.gov/sites/entrez?db=gene&cmd=Retrieve&dopt=Graphics&list_uids=1902) | -1.51 | -1.54 | -2.46 |
| [ELK1. member of ETS oncogene family](http://www.ncbi.nlm.nih.gov/sites/entrez?db=gene&cmd=Retrieve&dopt=Graphics&list_uids=1903) | -2.06 | -1.84 | -2.64 |
| [ELK4. ETS-domain protein (SRF accessory protein 1)](http://www.ncbi.nlm.nih.gov/sites/entrez?db=gene&cmd=Retrieve&dopt=Graphics&list_uids=9170) | -2.35 | -2.31 | -2.17 |
| [Fibroblast growth factor 2 (basic)](http://www.ncbi.nlm.nih.gov/sites/entrez?db=gene&cmd=Retrieve&dopt=Graphics&list_uids=9294) | -2.14 | -2.20 | -1.91 |
| [FBJ murine osteosarcoma viral oncogene homolog](http://www.ncbi.nlm.nih.gov/sites/entrez?db=gene&cmd=Retrieve&dopt=Graphics&list_uids=1906) | -5.03 | -1.95 | -1.38 |
| [GALANIN RECEPTOR 2](http://www.ncbi.nlm.nih.gov/sites/entrez?db=gene&cmd=Retrieve&dopt=Graphics&list_uids=1958) | 1.71 | 2.72 | 1.69 |
| [Glucagon receptor](http://www.ncbi.nlm.nih.gov/sites/entrez?db=gene&cmd=Retrieve&dopt=Graphics&list_uids=2002) | -1.28 | -1.06 | -1.85 |
| [Guanine nucleotide binding protein (G protein). q polypeptide](http://www.ncbi.nlm.nih.gov/sites/entrez?db=gene&cmd=Retrieve&dopt=Graphics&list_uids=2005) | -1.21 | -1.08 | -1.23 |
| [GNAS complex locus](http://www.ncbi.nlm.nih.gov/sites/entrez?db=gene&cmd=Retrieve&dopt=Graphics&list_uids=2247) | -1.21 | -1.10 | -1.39 |
| [Glutamate receptor. metabotropic 1](http://www.ncbi.nlm.nih.gov/sites/entrez?db=gene&cmd=Retrieve&dopt=Graphics&list_uids=2353) | 1.24 | 1.46 | 1.29 |
| [Glutamate receptor. metabotropic 2](http://www.ncbi.nlm.nih.gov/sites/entrez?db=gene&cmd=Retrieve&dopt=Graphics&list_uids=8811) | -1.49 | -1.39 | -2.95 |
| [Glutamate receptor. metabotropic 4](http://www.ncbi.nlm.nih.gov/sites/entrez?db=gene&cmd=Retrieve&dopt=Graphics&list_uids=2642) | 1.48 | 1.56 | 1.06 |
| [Glutamate receptor. metabotropic 5](http://www.ncbi.nlm.nih.gov/sites/entrez?db=gene&cmd=Retrieve&dopt=Graphics&list_uids=2776) | -1.31 | -1.10 | -1.54 |
| [Glutamate receptor. metabotropic 7](http://www.ncbi.nlm.nih.gov/sites/entrez?db=gene&cmd=Retrieve&dopt=Graphics&list_uids=2778) | -1.07 | -1.12 | 1.25 |
| [Intercellular adhesion molecule 1](http://www.ncbi.nlm.nih.gov/sites/entrez?db=gene&cmd=Retrieve&dopt=Graphics&list_uids=2911) | -1.07 | -1.12 | 1.25 |
| [Interleukin 1. beta](http://www.ncbi.nlm.nih.gov/sites/entrez?db=gene&cmd=Retrieve&dopt=Graphics&list_uids=2912) | -1.29 | -1.14 | -1.28 |
| [Interleukin 1 receptor. type I](http://www.ncbi.nlm.nih.gov/sites/entrez?db=gene&cmd=Retrieve&dopt=Graphics&list_uids=2914) | -2.58 | -1.72 | 1.06 |
| [Interleukin 1 receptor. type II](http://www.ncbi.nlm.nih.gov/sites/entrez?db=gene&cmd=Retrieve&dopt=Graphics&list_uids=2915) | -2.68 | -2.08 | -1.48 |
| [Interleukin 2](http://www.ncbi.nlm.nih.gov/sites/entrez?db=gene&cmd=Retrieve&dopt=Graphics&list_uids=2917) | -1.07 | -1.12 | 1.25 |
| [Jun proto-oncogene](http://www.ncbi.nlm.nih.gov/sites/entrez?db=gene&cmd=Retrieve&dopt=Graphics&list_uids=3383) | -1.85 | -1.82 | -1.48 |
| [Jun B proto-oncogene](http://www.ncbi.nlm.nih.gov/sites/entrez?db=gene&cmd=Retrieve&dopt=Graphics&list_uids=3553) | -2.56 | 1.17 | -1.13 |
| [Luteinizing hormone/choriogonadotropin receptor](http://www.ncbi.nlm.nih.gov/sites/entrez?db=gene&cmd=Retrieve&dopt=Graphics&list_uids=3554) | 1.01 | 1.36 | -2.55 |
| [Lysophosphatidic acid receptor 1](http://www.ncbi.nlm.nih.gov/sites/entrez?db=gene&cmd=Retrieve&dopt=Graphics&list_uids=7850) | -1.07 | -1.12 | 1.25 |
| [Lysophosphatidic acid receptor 2](http://www.ncbi.nlm.nih.gov/sites/entrez?db=gene&cmd=Retrieve&dopt=Graphics&list_uids=3558) | -1.07 | -1.12 | 1.25 |
| [MYC associated factor X](http://www.ncbi.nlm.nih.gov/sites/entrez?db=gene&cmd=Retrieve&dopt=Graphics&list_uids=3725) | 1.01 | -1.11 | 1.09 |
| [Matrix metallopeptidase 9 (gelatinase B. 92kDa gelatinase. 92kDa type IV collagenase)](http://www.ncbi.nlm.nih.gov/sites/entrez?db=gene&cmd=Retrieve&dopt=Graphics&list_uids=3726) | -1.55 | -1.67 | -1.33 |
| [V-myc myelocytomatosis viral oncogene homolog (avian)](http://www.ncbi.nlm.nih.gov/sites/entrez?db=gene&cmd=Retrieve&dopt=Graphics&list_uids=3973) | -1.07 | -1.12 | 1.25 |
| [Nitric oxide synthase 2. inducible](http://www.ncbi.nlm.nih.gov/sites/entrez?db=gene&cmd=Retrieve&dopt=Graphics&list_uids=4149) | -1.47 | -1.36 | -1.45 |
| [Opioid receptor. delta 1](http://www.ncbi.nlm.nih.gov/sites/entrez?db=gene&cmd=Retrieve&dopt=Graphics&list_uids=4318) | -1.48 | -1.27 | -1.28 |
| [Opioid receptor. kappa 1](http://www.ncbi.nlm.nih.gov/sites/entrez?db=gene&cmd=Retrieve&dopt=Graphics&list_uids=4609) | -2.02 | -1.91 | -1.93 |
| [3-phosphoinositide dependent protein kinase-1](http://www.ncbi.nlm.nih.gov/sites/entrez?db=gene&cmd=Retrieve&dopt=Graphics&list_uids=4843) | -1.39 | -1.14 | -1.11 |
| [Phosphoinositide-3-kinase. catalytic. gamma polypeptide](http://www.ncbi.nlm.nih.gov/sites/entrez?db=gene&cmd=Retrieve&dopt=Graphics&list_uids=4985) | -7.57 | -2.76 | -2.41 |
| [Protein kinase C. alpha](http://www.ncbi.nlm.nih.gov/sites/entrez?db=gene&cmd=Retrieve&dopt=Graphics&list_uids=4986) | -1.94 | -1.34 | 1.42 |
| [Prostaglandin D2 receptor (DP)](http://www.ncbi.nlm.nih.gov/sites/entrez?db=gene&cmd=Retrieve&dopt=Graphics&list_uids=5170) | -1.57 | -1.24 | -1.54 |
| [Prostaglandin-endoperoxide synthase 2 (prostaglandin G/H synthase and cyclooxygenase)](http://www.ncbi.nlm.nih.gov/sites/entrez?db=gene&cmd=Retrieve&dopt=Graphics&list_uids=5294) | -1.07 | -1.12 | 1.25 |
| [Parathyroid hormone 1 receptor](http://www.ncbi.nlm.nih.gov/sites/entrez?db=gene&cmd=Retrieve&dopt=Graphics&list_uids=5578) | -1.32 | -1.15 | -1.19 |
| [Regulator of G-protein signaling 2. 24kDa](http://www.ncbi.nlm.nih.gov/sites/entrez?db=gene&cmd=Retrieve&dopt=Graphics&list_uids=5729) | -1.15 | 2.03 | 2.58 |
| [Rhodopsin](http://www.ncbi.nlm.nih.gov/sites/entrez?db=gene&cmd=Retrieve&dopt=Graphics&list_uids=5743) | 2.03 | 2.93 | 1.79 |
| [Sphingosine-1-phosphate receptor 1](http://www.ncbi.nlm.nih.gov/sites/entrez?db=gene&cmd=Retrieve&dopt=Graphics&list_uids=5745) | -1.89 | -1.52 | -1.42 |
| [Sphingosine-1-phosphate receptor 2](http://www.ncbi.nlm.nih.gov/sites/entrez?db=gene&cmd=Retrieve&dopt=Graphics&list_uids=5997) | -1.13 | 1.10 | -1.25 |
| [Sphingosine-1-phosphate receptor 3](http://www.ncbi.nlm.nih.gov/sites/entrez?db=gene&cmd=Retrieve&dopt=Graphics&list_uids=6010) | -2.55 | -2.26 | -1.25 |
| [Secretin receptor](http://www.ncbi.nlm.nih.gov/sites/entrez?db=gene&cmd=Retrieve&dopt=Graphics&list_uids=6344) | -1.07 | -1.12 | 1.25 |
| [Serpin peptidase inhibitor. clade E (nexin. plasminogen activator inhibitor type 1). member 1](http://www.ncbi.nlm.nih.gov/sites/entrez?db=gene&cmd=Retrieve&dopt=Graphics&list_uids=5054) | -2.22 | -1.27 | -3.14 |
| [Suppressor of cytokine signaling 1](http://www.ncbi.nlm.nih.gov/sites/entrez?db=gene&cmd=Retrieve&dopt=Graphics&list_uids=8651) | -1.66 | -1.77 | -1.60 |
| [Tumor necrosis factor](http://www.ncbi.nlm.nih.gov/sites/entrez?db=gene&cmd=Retrieve&dopt=Graphics&list_uids=7124) | -1.78 | -2.21 | -2.79 |
| [Thyroid stimulating hormone receptor](http://www.ncbi.nlm.nih.gov/sites/entrez?db=gene&cmd=Retrieve&dopt=Graphics&list_uids=7253) | -1.22 | -1.52 | -2.35 |
| [Uncoupling protein 1 (mitochondrial. proton carrier)](http://www.ncbi.nlm.nih.gov/sites/entrez?db=gene&cmd=Retrieve&dopt=Graphics&list_uids=7350) | 1.41 | 2.12 | -1.08 |
| [Vascular cell adhesion molecule 1](http://www.ncbi.nlm.nih.gov/sites/entrez?db=gene&cmd=Retrieve&dopt=Graphics&list_uids=7412) | -1.07 | -1.12 | 1.25 |
| [Vascular endothelial growth factor A](http://www.ncbi.nlm.nih.gov/sites/entrez?db=gene&cmd=Retrieve&dopt=Graphics&list_uids=7422) | -1.21 | -1.25 | -1.05 |
| [Tyrosine 3-monooxygenase/tryptophan 5-monooxygenase activation protein. zeta polypeptide](http://www.ncbi.nlm.nih.gov/sites/entrez?db=gene&cmd=Retrieve&dopt=Graphics&list_uids=7534) | -1.99 | -1.65 | -1.92 |
| [Actin. beta](http://www.ncbi.nlm.nih.gov/sites/entrez?db=gene&cmd=Retrieve&dopt=Graphics&list_uids=567) | -1.23 | -1.07 | -1.68 |
| [Beta-2-microglobulin](http://www.ncbi.nlm.nih.gov/sites/entrez?db=gene&cmd=Retrieve&dopt=Graphics&list_uids=3251) | -1.17 | -1.04 | -1.21 |
| [Glyceraldehyde-3-phosphate dehydrogenase](http://www.ncbi.nlm.nih.gov/sites/entrez?db=gene&cmd=Retrieve&dopt=Graphics&list_uids=23521) | 1.00 | 1.00 | 1.00 |
| [Hypoxanthine phosphoribosyltransferase 1](http://www.ncbi.nlm.nih.gov/sites/entrez?db=gene&cmd=Retrieve&dopt=Graphics&list_uids=2597) | 1.08 | 1.12 | -1.17 |
| [Ribosomal protein. large. P0](http://www.ncbi.nlm.nih.gov/sites/entrez?db=gene&cmd=Retrieve&dopt=Graphics&list_uids=60) | -1.59 | -1.22 | -2.00 |
